# Netrin-1 induces the anti-apoptotic and pro-survival effects of B-ALL cells through the Unc5b-MAPK axis

**DOI:** 10.1186/s12964-022-00935-y

**Published:** 2022-08-16

**Authors:** Lan Huang, Xizhou An, Yao Zhu, Kainan Zhang, Li Xiao, Xinyuan Yao, Xing Zeng, Shaoyan Liang, Jie Yu

**Affiliations:** 1 Department of Hematology and Oncology, Children’s Hospital of Chongqing Medical University, 136 Zhongshanerlu, Yuzhong district, Chongqing, 400014 China; 2 National Clinical Research Center for Child Health and Disorders, Ministry of Education Key Laboratory of Child Development and Disorders, Chongqing Key Laboratory of Pediatrics, Chongqing, China; 3 Pediatric Research Institute, Children’s Hospital of Chongqing Medical University, Chongqing, China

**Keywords:** Netrin-1, B-cell acute lymphoblastic leukemia, Unc5b, FAK, MAPK, Apoptosis

## Abstract

**Background:**

B-cell acute lymphoblastic leukemia (B-ALL) comprises over 85% of all acute lymphoblastic leukemia (ALL) cases and is the most common childhood malignancy. Although the 5 year overall survival of patients with B-ALL exceeds 90%, patients with relapsed or refractory B-ALL may suffer from poor prognosis and adverse events. The axon guidance factor netrin-1 has been reported to be involved in the tumorigenesis of many types of cancers. However, the impact of netrin-1 on B-ALL remains unknown.

**Methods:**

The expression level of netrin-1 in peripheral blood samples of children with B-ALL and children without neoplasia was measured by enzyme-linked immunosorbent assay (ELISA) kits. Then, CCK-8 cell proliferation assays and flow cytometric analysis were performed to detect the viability and apoptosis of B-ALL cells (Reh and Sup B15) treated with exogenous recombinant netrin-1 at concentrations of 0, 25, 50, and 100 ng/ml. Furthermore, co-immunoprecipitation(co-IP) was performed to detect the receptor of netrin-1. UNC5B expression interference was induced in B-ALL cells with recombinant lentivirus, and then CCK-8 assays, flow cytometry assays and western blotting assays were performed to verify that netrin-1 might act on B-ALL cells via the receptor Unc5b. Finally, western blotting and kinase inhibitor treatment were applied to detect the downstream signaling pathway.

**Results:**

Netrin-1 expression was increased in B-ALL, and netrin-1 expression was upregulated in patients with high- and intermediate-risk stratification group of patients. Then, we found that netrin-1 induced an anti-apoptotic effect in B-ALL cells, implying that netrin-1 plays an oncogenic role in B-ALL. co-IP results showed that netrin-1 interacted with the receptor Unc5b in B-ALL cells. Interference with UNC5B was performed in B-ALL cells and abolished the antiapoptotic effects of netrin-1. Further western blotting was applied to detect the phosphorylation levels of key molecules in common signaling transduction pathways in B-ALL cells treated with recombinant netrin-1, and the FAK-MAPK signaling pathway was found to be activated. The anti-apoptotic effect of netrin-1 and FAK-MAPK phosphorylation was abrogated by UNC5B interference. FAK inhibitor treatment and ERK inhibitor treatment were applied and verified that the FAK-MAPK pathway may be downstream of Unc5b.

**Conclusion:**

Taken together, our findings suggested that netrin-1 induced the anti-apoptotic effect of B-ALL cells through activation of the FAK-MAPK signaling pathway by binding to the receptor Unc5b.

**Video Abstract**

**Supplementary Information:**

The online version contains supplementary material available at 10.1186/s12964-022-00935-y.

## Background

B-cell lymphoblastic leukemia (B-ALL) comprises over 85% of all ALL [1]which is the most common childhood malignancy [2]. In recent years, although the cure rate for B-ALL has been substantially raised due to continuous optimization of the treatment scheme, the relapse rate remains 10%-15% in children with B-ALL [3, 4]. The relapse of B-ALL remains the leading cause of death, and contributes to approximately 60% of the mortality caused by the disease [5].

Anti-apoptosis is one of the usual causes of B-ALL relapse [6]. The Bcl-2 family, Bak and Bax, which are located in mitochondria, are apoptosis related proteins responsible for regulating pro-and anti- apoptotic effect. The drop in the Bax/Bcl-2 ratio was associated with relapse and remission failure in B-ALL [7, 8]. Moreover, overexpression of Bcl-2 family proteins is one chemoresistant mechanism [6]. However, the underlying mechanism through which Bcl-2 is upregulated in B-ALL is still not clear. Finding a key regulator which induces the Bcl-2 upregulation and anti-apoptosis effects in B-ALL could make sense to expand the potential application of Bcl-2 related regimens for relapsed or refractory B-ALL.Netrin-1 (encoded by the *NTN1* gene), belongs to a family of secreted laminin-related proteins, and has been proven to play an essential role in axon guidance during the development of the nervous system [9–11]. Overexpression of netrin-1 has been observed in many types of advanced cancers, such as neuroblastoma, colorectal cancer, pancreatic cancer and hepatic cancer [12]. Netrin-1 has been reported to act as a novel promotor of cancer cell survival and invasiveness by inhibiting apoptosis through its receptors, such as DCC, Unc5 homologues (Unc5a, Unc5b, Unc5c and Unc5d) and Neo1 [13–15]. However, whether netrin-1 and its receptor participate in the anti-apoptosis of B-ALL remains poorly understood. In the present study, we investigated the role of netrin-1 and its receptors in B-ALL progression. We aimed to clarify the role of netrin-1 and its receptor in the anti-apoptosis effect of B-ALL cells, and furthermore, we investigated the signal transduction pathway involved in this process.

## Materials and methods

### Samples and patients

Peripheral blood samples were obtained from 50 children diagnosed with B-ALL and 27 children with nonneoplastic disorders who were hospitalized at Children’s Hospital affiliated to Chongqing Medical University (CHCMU) from December, 2019 to October, 2020. No medical therapy was administered before peripheral blood collection. B-ALL was diagnosed according to the WHO morphological, immunophenotypic, cytogenetic, and molecular (MICM) criteria for classifying hematopoietic and lymphoid tissue tumors 2008 and 2016, respectively [16, 17]. All children received risk stratification evaluation following the Chinese Children Cancer Group (CCCG) 2015 ALL chemotherapy protocol [18, 19]. Data regarding their clinical manifestations were obtained from the standard electronic medical records system of the CHCMU. Written informed consent was obtained from all patients or guardians. The use of peripheral blood samples was approved by the Ethics Committee of Children’s Hospital of Chongqing Medical University. The Ethics Committee permission number is No.2019–253.

### Antibodies and reagents

The recombinant netrin-1 protein used to treat B-ALL cells was purchased from R&D systems. The FAK inhibitor PF-573228 (HY-10461, MCE) and the ERK inhibitor Magnolin (HY-N1314, MCE) were used to study FAK-MAPK signaling. Anti-His-tag antibody (#12698, Cell Signaling Technology) and anti-normal IgG (A00002, ZENBIO) were used for co-immunoprecipitation. Anti-Neogenin (ab183511,Abcam), Itgβ1 (WL01615, Wanleibio), Itgα3(ab190731, Abcam), anti-Itgβ4(ab182120, Abcam), anti-Unc5b (#13851, Cell Signaling Technology) and anti-Unc5a (22068-1-AP, Proteintech) were used for western blotting. Anti-PCNA(#13110, Cell Signaling Technology), anti-CDK4 (#12790, Cell Signaling Technology), anti-cyclinE2 (#4132, Cell Signaling Technology), anti-Bcl-2 (60178-1-Ig, proteintech), anti-Bax (60267-1-Ig, proteintech), and anti-Gapdh(390035, ZENBIO), anti-FAK (#71,433, Cell Signaling Technology), anti-p-FAK (#8556, Cell Signaling Technology, Danvers, MA, USA), anti-c-Raf (#53,745, Cell Signaling Technology), anti-pc-Raf (#9421, Cell Signaling Technology), anti-MEK1/2 (#9126,Cell Signaling Technology), anti-pMEK1/2 (#9154,Cell Signaling Technology), anti-ERK1/2 (#4695, Cell Signaling Technology), anti-pERK1/2 (#4370, Cell Signaling Technology), anti-AKT (#4691, Cell Signaling Technology), anti-pAKT (#4060, Cell Signaling Technology), anti-P85 (#4257, Cell Signaling Technology), anti-pP85 (#4228, Cell Signaling Technology), anti-P50 (#3035, Cell Signaling Technology), anti-pP50 (#4806, Cell Signaling Technology), anti-P65 (#8242, Cell Signaling Technology) and anti-pP65 (#3303, Cell Signaling Technology) were used as primary antibodies in western blotting assays. Horseradish peroxidase (HRP) goat anti-rabbit IgG (511203, ZENBIO) and HRP conjugated rabbit anti-mouse IgG (511103, ZENBIO) were used as the secondary antibodies for western blotting.

### Enzyme-linked immunosorbent assay (ELISA)

The serum concentrations of netrin-1 were measured by using enzyme-linked immunosorbent assay (ELISA) kits (Elabscience, Wuhan, China). Briefly, netrin-1 standards and samples were added to antibody-coated 96-well microtiter plates and incubated at 37 °C for 90 min. Then biotinylated detection antibody specific for netrin-1 was added to each well and incubated for another 1 h. The plates were then washed and incubated with avidin conjugated to horseradish peroxidase for 30 min. Tetramethylbenzidine was the substrate for color development, and the reaction was inhibited by adding stop solution. Finally, the absorbance was measured at 450 nm. Serum netrin-1 concentrations were measured by comparing the OD values of samples to the standard curve. All measurements were made in duplicate.

### Cell lines and cell culture

The human B-ALL cell lines SUP-B15 (ATCC®CRL-1929™) and REH (ATCC®CRL-8286™) were obtained from an authorized distributor of ATCC in China (Zhongyuan Inc.). The cells were cultured in RPMI 1640 medium (GIBCO, Waltham, MA, USA) with 10% fetal bovine serum (Cat no. 10100147, GIBCO, Waltham, MA, USA), 100U/mL streptomycin and 100 U/mL penicillin. B-ALL cells were incubated at 37 °C in a humidified atmosphere containing 5% CO_2_. The medium was changed, and the cells were passaged every 2–3 days according to the instructions.

### CCK-8 cell growth detection

The proliferation of B-ALL cells was detected using Cell Counting Kit-8 (CCK-8) (MCE, USA). Cells were seeded in a 96-well plate, with 8000 cells per well and incubated for 3 days. CCK-8 solution (10 μL) was added to each plate well at specific time points (0, 24, 48, 72 h), and the cells were incubated at 37 °C for 3 h. Then the absorbance was measured at 450 nm. Each assay was performed in triplicate.

### Transwell assay

Transwell assays were performed using Matrigel-coated Transwell chambers (Corning Costar, USA) with an 8.0 μm pore size. B-ALL cells (2 × 105/mL) were seeded into the upper compartment in 200 μL of serum-free medium. The lower chambers were added to various concentrations of netrin-1 ranging from 0 to 200 ng/ml in 800 μL medium containing 10% FBS. After incubation at 37 °C for 24 h, non-migrated cells on the upper compartment were removed by a cotton swab, and cells in the lower chambers were fixed with formaldehyde and stained with 0.5% crystal violet. The stained cells were then counted under a TE2000-U inverted fluorescence microscope (Nikon Inc., Chiyoda-ku, Tokyo, Japan). Each assay was performed in triplicate.

### Detection of apoptosis by flow cytometry

Apoptotic cells were stained with Annexin V-7AAD/APC apoptosis detection kits (KeyGEN Biotech, Jiangsu, China). All procedures were performed according to manufacturer's instructions. Stained cells were analyzed using a flow cytometer (BD Biosciences, San Jose, CA, USA). Apoptotic cells were characterized as cells with high fluorescence levels of Annexin V and low levels of 7AAD (lower right). Each assay was performed in triplicate.

### Quantitative real-time RT–PCR

Total RNA was extracted from cultured B-ALL cells using TRIzol (Invitrogen, Waltham, MA, USA), following the manufacturer’s instructions. The RNA concentration was examined by a NanoDrop™ 2000UV–Vis Spectrophotometer (ThermoFisher, Waltham, MA, USA). Complementary DNA (cDNA) were produced by using the TaKaRa RNA PCR Kit (TaKaRa Bio, Kusatsu, Shiga, Japan). The cDNA was amplified by SYBR Green real-time qPCR Kit (TaKaRa Bio) according to the manufacturer’s instructions. The expression levels of mRNA were measured by 2^−ΔCt^ method. β-actin expression levels were used as internal control. The primers used in real-time (qRT-PCR) are shown in Additional file 1: Table S1. Each assay was performed in triplicate.

### Co-immunoprecipitation

The cells were treated with recombinant netrin-1 in concentration ladder of 0, 25, 50 and 100 ng/ml for 24 h. Then total protein was extracted from REH cells and SUP-B15 cells using cell lysis kit (ThermoFisher Waltham, WA, USA). A BCA protein assay kit (Beyotime, Shanghai, China) was used to determine the protein concentrations. Then the acquired proteins (500–1000 ng) were incubated with specific antibodies or normal IgG at 4 °C overnight. A total of 25 μL of A/G magnetic beads was added and incubated with the immune complexes for 1 h.Finally, the protein was eluted from the beads for western blotting.

### Western blotting

The cells were treated with recombinant netrin-1 in concentration ladder of 0, 25, 50 and 100 ng/ml for 24 h. Then the cells were lysed and dissolved in a cell lysis kit (BestBio, Shanghai, China). A total of 20 ng of protein was resolved using 6% SDS–PAGE gels and transferred to PVDF membranes (Millipore, USA). The membranes were blocked in 5% skim milk for 1 h before being incubated with primary antibodies (1:1000) overnight at 4 °C. The membranes were then washed with TBST (Tris buffered saline with 0.1% Tween-20), and incubated with secondary antibodies (1:5000) for 1 h. Protein bands were visualized using an ECL Kit (Bio–Rad Laboratories, Hercules, CA, USA).

### RNA interference to the UNC5B

UNC5B RNA interference (RNAi) lentivirus expressed the sequence [20]: 5′-gatccGCCACACAGATCTACTTCAATTCAAGAGATTGAAGTAGATCTGTGTGGTTTTTTg-3′, which was designed from UNC5B cDNA sequence NM_001244889.1. UNC5B shRNA lentivirus (shUNC5B) and non-sense scramble shRNA lentivirus were synthesized by (GENE, Shanghai, China). When REH cells and SUP-B15 cells confluence reached 60%-70% confluence, the cells were transfected with LV-shUNC5B or LV-shctrl at 37 °C for 48 h. Generally, GFP expression was detectable 72 h following infection. Puromycin was used to select puromycin resistant cells, and then screened out cells those were successfully infected. Furthermore, UNC5B expression was measured by Western blotting.

### Statistical analysis

Data were statistically analyzed using SPSS version 23.0. The data were presented as the mean and the standard error of the mean (SEM) unless indicated otherwise. The statistical significance of differences between two sets of data was evaluated by the Mann–Whitney U test. Differences between groups were examined by one-way or two-way analysis of variance (ANOVA) with multiple comparisons, followed by Bonferroni post hoc test. *P* value < 0.05 was considered statistically significant.

## Results

### The expression level of netrin-1 in B-ALL patients

**Fig. 1:**
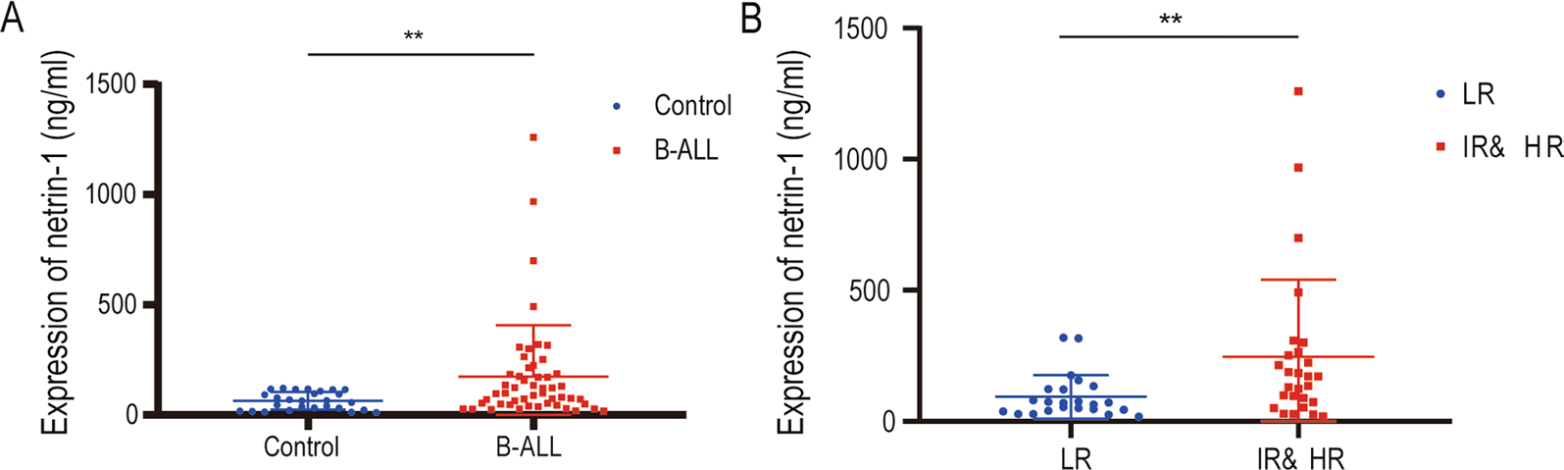
Netrin-1 expression was increased in B-ALL patients. **A** Enzyme-linked immunosorbent assay for measuring netrin-1 in B-ALL patients and patients with nonneoplastic disorders(ctrl) (B-ALL *n* = 50, ctrl *n* = 27, **P < 0.01). **B** ELISA for measuring netrin-1 in B-ALL patients with high and intermediate risk stratification and B-ALL patients with low risk stratification (LR *n* = 23, IR & HR *n* = 27, *P < 0.05, **P < 0.01)

To explore the role of netrin-1 in B-ALL progression, we collected peripheral blood samples obtained from 50 children with B-ALL and 27 children with nonneoplastic disorders and measured serum netrin-1 concentrations using ELISA (Fig. 1A). The results revealed that serum netrin-1 concentrations were significantly upregulated in B-ALL group compared with control group (*P* < 0.01). Furthermore, we grouped B-ALL patients according to different clinical indicators and compared serum netrin-1 concentrations in different subgroups (Fig. 1B and Table 1). Serum netrin-1 concentrations were significantly higher in patients with high and intermediate risk stratification than in patients with low risk stratification (*P* < 0.01).

**Table 1:** Netrin-1 expression levels in B-ALL children with different risk stratifications

Risk stratification	*n*	Netrin-1(ng/ml)	*P*
LR	23	70.08	0.0089**
IR HR	27	171.6	

**P < 0.01

### Netrin-1 induced anti-apoptotic effect of B-ALL cells

**Fig. 2:**
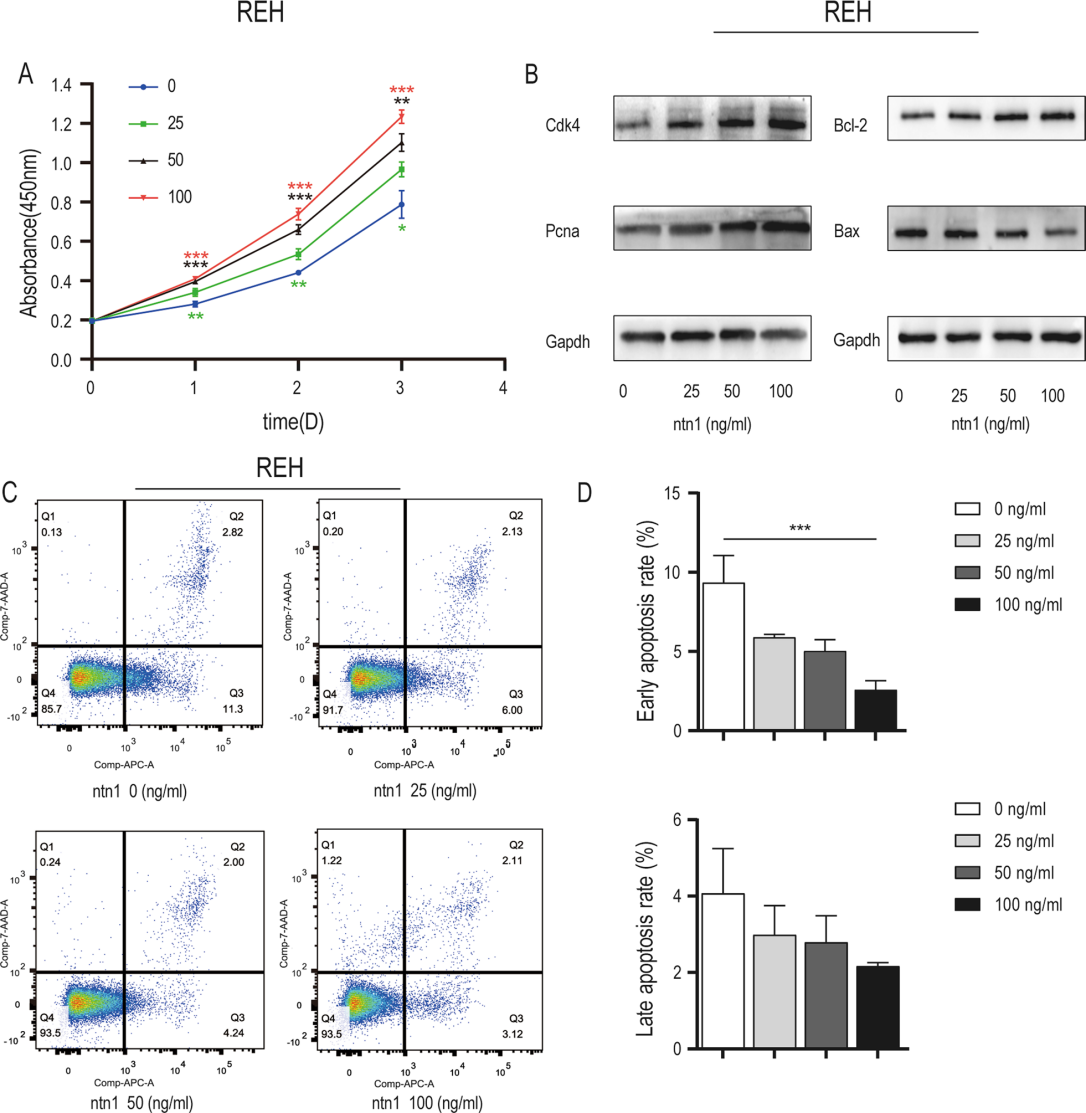
Netrin-1 induced the anti-apoptotic effect of REH cells. **A** The 450 nm absorbance of REH cells treated with exogenous recombinant netrin-1 in concentration ladder of 0,25,50,100 ng/ml in 3 days. (*n* = 3, *P < 0.05, **P < 0.01, ***P < 0.001**)**.** B** The expression of CDK4, PCNA, Bcl-2 and Bax in REH cells treated with exogenous netrin-1 in concentration ladder (0,25,50,100 ng/ml, 24 h) was detected by western blotting. The expression of Gapdh was applied as an internal control. **C** Flow cytometric analysis of the apoptotic ratio of REH cells treated with exogenous recombinant netrin-1 in concentration ladder of 0, 25, 50 and 100 ng/ml after 24 h. The early apoptotic cells were marked with positive Annexin V staining (APC-A) and negative 7-AAD staining. **D** Column graph of the early and late apoptosis ratio of REH cells treated with exogenous recombinant netrin-1 in concentration ladder of 0,25,50 and 100 ng/ml after 24 h detected by flow cytometric analysis (*n* = 3, ***P < 0.001)

To further explore how netrin-1 promoted B-ALL progression, we assessed the role of netrin-1 in two cultured B-ALL cell lines, REH and SUP-B15. We incubated different concentrations of recombinant netrin-1 with B-ALL cells to examine the effects of netrin-1 on cell proliferation. The CCK-8 assay (Fig. 2A, Additional file 2: Fig S2A) revealed that the survival rate of B-ALL cells treated with recombinant netrin-1 was higher than that of the control group (no recombinant netrin-1 treatment). Recombinant netrin-1 treatment reduced the growth rate of B-ALL cells in a dose-dependent manner, and the most effective concentration was 100 ng/ml for REH cells and 50 ng/ml for SUP-B15 cells. Western blotting was performed to detect PCNA, which is a nuclear protein closely related to cell proliferation [21], CDK4, which is key to cell cycle [22], Bcl-2, which is an antiapoptotic protein [23], and Bax, which is a death-promoting protein inducing mitochondria-dependent programed cell death [24]. The western blotting assay results (Fig. 2B, Additional file 2: Fig S2B). showed that CDK4, PCNA and Bcl-2 increased after recombinant netrin-1 treatment. However, the protein levels of Bax decreased.The increase in CDK4, PCNA and Bcl-2 expression and decrease in Bax expression are associated with an increase in cell proliferation. The apoptosis rate of B-ALL cells was measured by Flow cytometry, which was consistent with the results of the CCK8 result (Fig. 2C, D, Additional file 2: Fig S2C, D). We found that recombinant netrin-1 reduced the cell apoptosis rate compared with the blank control group. Collectively, these results showed that netrin-1 induced the anti-apoptotic effect of B-ALL cells in vitro.

### Netrin-1 did not influence the migration of B-ALL cells

Netrin-1 signaling was found to participate in the invasion and migration of malignant cells in multiple types of cancer [25–27]. Therefore, we assessed the role of netrin-1 in B-ALL cell motility by Transwell migration assays in this study (Additional file 3: Fig S1). However, the average numbers of migrated B-ALL cells showed no significant difference between all netrin-1 treating groups and the blank control group. These results suggested that Netrin-1 treatment has no significant influence on B-ALL cell motility.

### *Netrin-1 suppressed apoptosis of B-ALL cells *via* Unc5b receptor*

**Fig. 3:**
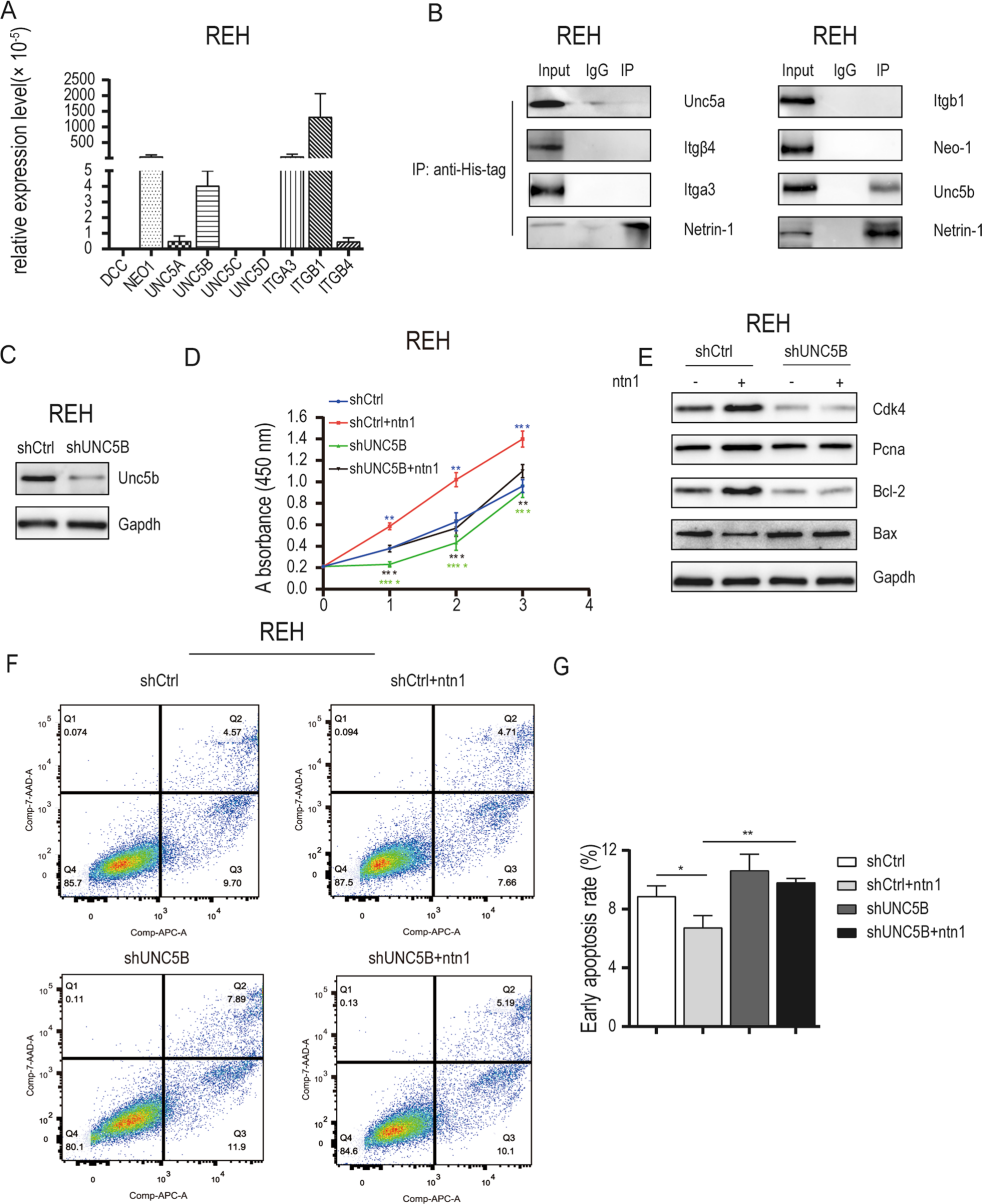
Netrin-1 induced the anti-apoptotic effect of REH cells through the Unc5b receptor. **A** Real-time PCR analysis of the expression of netrin-1 receptor in REH cells. ACTIN was used as an internal control. **B** An anti-His-tag antibody was used to pull down the histagged netrin-1 protein after exogenous recombinant netrin-1 treatment, followed by immunoblotting analysis of the receptor and netrin-1 levels in the precipitation. **C** The expression of Unc5b was efficiently decreased following transfection with UNC5B interference lentivirus in REH cells. **D** The 450 nm absorbance of the 3 day growth curve of shCtrl cells, shCtrl cells treated with netrin-1(100 ng/ml), shUNC5B cells and shUNC5B cells treated with netrin-1(*n* = 3, **P < 0.01, ***P < 0.001, ****P < 0.0001). **E** The expression levels of CDK4, PCNA, Bcl-2 and Bax in shCtrl cells, shCtrl cells treated with netrin-1 alone (100 ng/ml, 24 h), shUNC5B cells and shUNC5B cells treated with netrin-1. The expression level was detected by western blotting. The expression of Gapdh was applied as an internal control. **F** Flow cytometric analysis of the apoptotic ratio of shCtrl cells, shCtrl cells treated with netrin-1 alone (100 ng/ml, 24 h), shUNC5B cells and shUNC5B cells treated with netrin-1. **G** Column graph of the early apoptotic ratio of shCtrl cells, shCtrl cells treated with netrin-1 alone, shUNC5B cells and shUNC5B cells treated with netrin-1. (*n* = 3, *P < 0.05, **P < 0.01)

Netrin-1 played its role by interacting with its receptors on the cell membrane. To investigate the mechanism by which netrin-1 suppressed the apoptosis of B-ALL cells, the expression of netrin-1 receptors was detected in B-ALL cell lines. Six potential receptors —neogenin, integrin α3, integrin β1, integrin β4, uncoordinated − 5a (Unc5a) and uncoordinated − 5b (Unc5b) were expressed in both cell lines, while the expression of DCC and other uncoordinated family members was at a relatively lower or undetectable level in both two cell lines (Fig. 3A, Additional file 4: Fig S3A). Co-immunoprecipitation assays were performed (Fig. 3B, Additional file 4: Fig S3B) and the results showed that His-tagged recombinant netrin-1 precipitated with Unc5b instead of neognin, integrin α3, integrin β1, integrin β4 or Unc5a. Furthermore, UNC5B expression interference was induced in B-ALL cells with recombinant lentivirus. Following infection with the UNC5B-targeting lentivirus, UNC5B expression was measured in REH cells and SUP-B15 cells by western blotting (Fig. 3C, Additional file 4: Fig S3C). Unc5b expression was markedly decreased, indicating that transfection was successful. The CCK-8 assays (Fig. 3D; Additional file 4: Fig S3D) revealed that UNC5B interference significantly reduced the proliferation ability of B-ALL cells treated with recombinant netrin-1. Flow cytometry assays to detect apoptosis were performed (Fig. 3F, G; Additional file 4: Fig S3F, 3G) and the results showed that UNC5B interference increased the apoptosis rate of B-ALL cells treated with recombinant netrin-1 compared with the control group with recombinant netrin-1 treatment and prohibited the anti-apoptotic effect of netrin-1. Western blotting assays (Fig. 3E; Additional file 4: Fig S3E) revealed that UNC5B interference in B-ALL cells treated with recombinant netrin-1 decreased the protein levels of CDK4, PCNA and Bcl-2 respectively and increased the protein levels of Bax compared with the control group treated with recombinant netrin-1.These results revealed that Unc5b might be the major receptor for netrin-1 to induce the anti-apoptotic effect of B-ALL cells.

### Netrin-1/Unc5b activates FAK-MAPK pathway

**Fig. 4:**
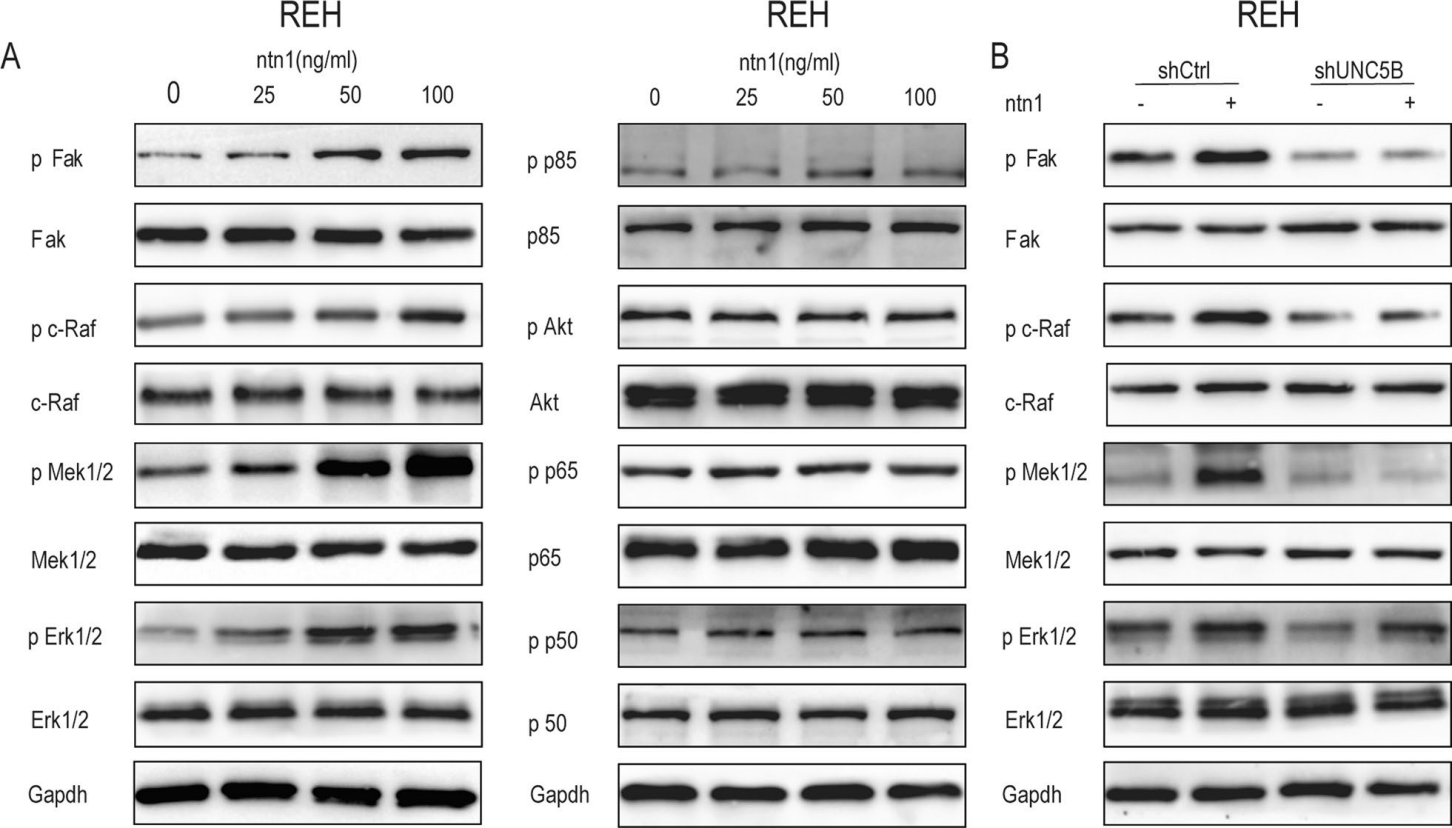
Netrin-1 increased the phosphorylation of FAK-MAPK pathway in REH cells. **A** The total expression level and phosphorylation level of FAK, c-Raf, Mek1/2,Erk1/2, p85, Akt, p65 and P50 in REH cells treated with exogenous netrin-1 in concentration ladder (0,25,50,100 ng/ml,30 min). The expression level was detected by western blotting. The expression level of Gapdh was applied as internal control. **B** Interference of UNC5B expression decreased the phosphorylation of FAK-MAPK pathway in REH cells treated with netrin-1. The total protein expression level and phosphorylation level of FAK, c-Raf, Mek1/2 and Erk1/2 in shCtrl cells, shCtrl cells treated with netrin-1 alone (100 ng/ml, 30 min), shUNC5B cells and shUNC5B cells treated with netrin-1 were detected by western blotting assay. The expression level of Gapdh was applied as an internal control

To explore the pathway downstream of netrin-1 in B-ALL cells, we performed Western blotting (Fig. 4A, Additional file 5: Fig S4A) to detect the phosphorylation levels of key molecules in common signaling transduction pathways in tumors [28–31]. The western blotting results showed that the phosphorylation levels of Fak, c-Raf, Mek and Erk were increased, suggesting that FAK-MAPK signaling cascades phosphorylation was enhanced after exogenous recombinant netrin-1 treatment.

**Fig. 5:**
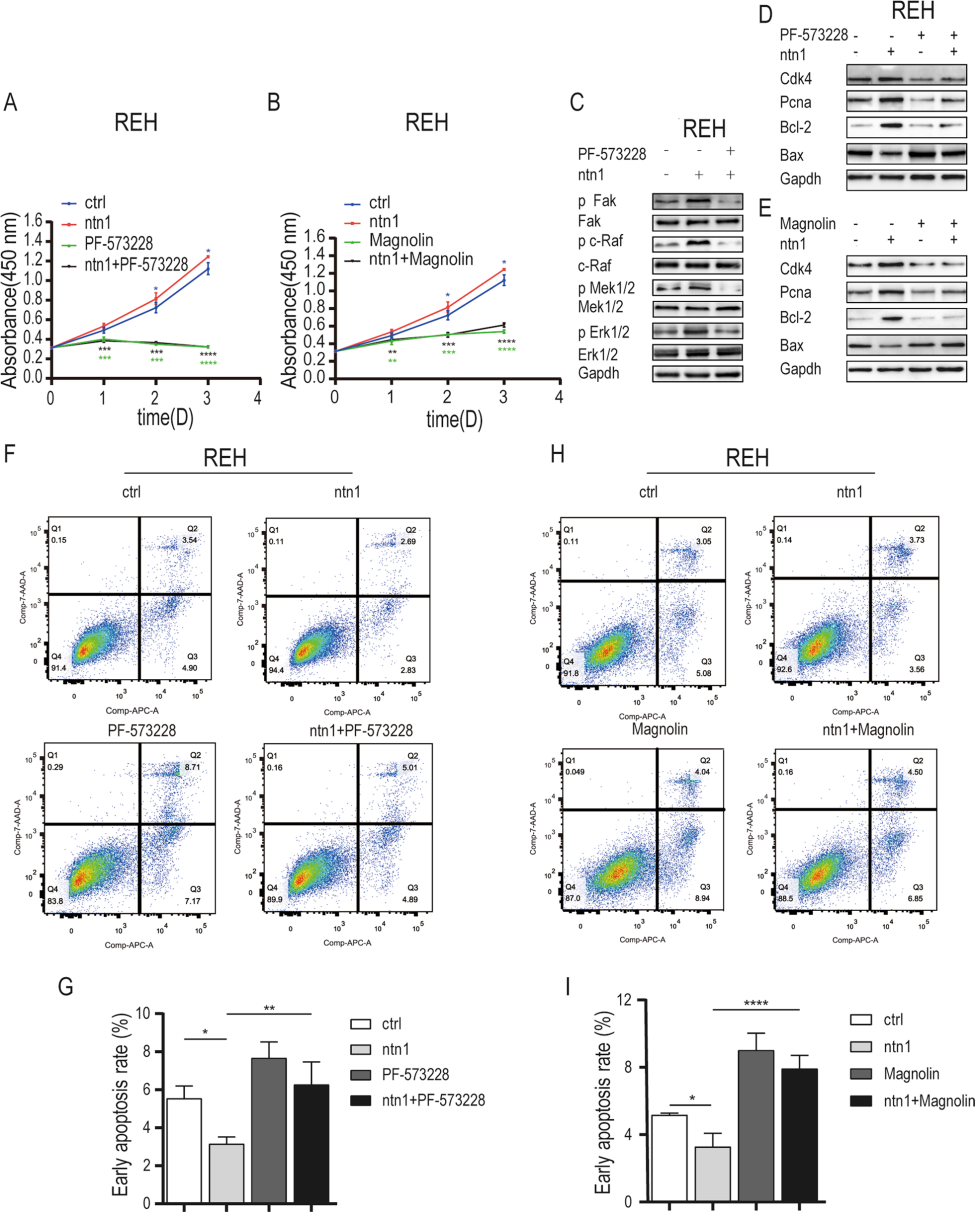
Inhibition of FAK and Erk 1/2 could reduce REH cells survival. **A** The 450 nm absorbance of the 3 day growth curve of REH cells (ctrl), REH cells treated with netrin-1 alone(100 ng/ml), REH cells treated with PF-573228 alone (20 nM) and REH cells co-treated with netrin-1 and PF-573228(*n* = 3, ***P < 0.001, ****P < 0.0001). **B** The 450 nm absorbance of the 3 day growth curve of REH cells (ctrl), REH cells treated with netrin-1 alone (100 ng/ml), REH cells treated with Magnolin alone (87 nM) and REH cells co-treated with netrin-1and Magnolin (*n* = 3, *P < 0.05, **P < 0.01, ***P < 0.001, ****P < 0.0001). **C** The total protein expression level and phosphorylation level of FAK, c-Raf, Mek1/2 and Erk1/2 in REH cells treated with netrin-1 alone (100 ng/ml, 24 h) and REH cells co-treated with netrin-1 and PF-573228(20 nM, 24 h). The expression level of Gapdh was applied as an internal control. **D** The expression levels of CDK4, PCNA, Bcl-2 and Bax in REH cells (ctrl), REH cells treated with netrin-1 alone (100 ng/ml, 24 h), REH cells treated with PF-573228 alone (20 nM, 24 h) and REH cells co-treated with netrin-1 and PF-573228 were detected by western blotting. The expression level of Gapdh was applied as an internal control. **E** The expression level of CDK4, PCNA, Bcl-2 and Bax in REH cells (ctrl), REH cells treated with netrin-1 alone (100 ng/ml, 24 h), REH cells treated with Magnolin alone (87 nM, 24 h) and REH cells co-treated with netrin-1 and Magnolin were detected by western blotting. The expression level of Gapdh was applied as an internal control. **F** Flow cytometric analysis of apoptotic ratio of REH cells (ctrl), REH cells treated with netrin-1 alone (100 ng/ml, 24 h), REH cells treated with PF-573228 alone (20 nM, 24 h) and REH cells co-treated with netrin-1 and PF-573228. **G** Column graph of the early apoptotic ratio of REH cells (ctrl), REH cells treated with netrin-1 alone, REH cells treated with PF-573228 alone and REH cells co-treated with netrin-1 and PF-573228 (*n* = 3, *P < 0.05, **P < 0.01). **H** Flow cytometric analysis of apoptotic ratio of REH cells (ctrl), REH cells treated with netrin-1 alone (100 ng/ml, 24 h), REH cells treated with Magnolin (87 nM, 24 h) and REH cells co-treated with netrin-1 and Magnolin. **I** Column graph of the early apoptotic ratio of REH cells (ctrl), REH cells treated with netrin-1 alone, REH cells treated with Magnolin alone and REH cells co-treated with netrin-1 and Magnolin (*n* = 3, *P < 0.05, ****P < 0.0001).

To confirm the regulatory effect of netrin-1 on FAK-MAPK pathway, we detected the phosphorylation levels of the FAK-MAPK signaling pathway in B-ALL cells following UNC5B interference. The western blotting results (Fig. 4B, Additional file 5: Fig S4B) showed that UNC5B interference aborted the activation effect of FAK-MAPK signaling cascades by netrin-1 in B-ALL cells. Furthermore, CCK-8 cell proliferation assays and flow cytometry assays for apoptosis were conducted in B-ALL cells treated with the FAK inhibitor, PF-573228. The results of CCK-8 assays (Fig. 5A, Additional file 6: Fig S5A) showed that PF-573228 decreased the proliferation of B-ALL cells treated with netrin-1. The apoptosis rates of both REH cells and SUP-B15 cells treated with netrin-1 alone were significantly increased after treatment with PF-573228 and netrin-1 (Fig. 5F, G; Additional file 6: Fig S5F, 5G), which was similar to UNC5B interference. The Western blotting assays (Fig. 5D, Additional file 6: Fig S5D) showed that the protein levels of CDK4, PCNA and Bcl-2 decreased while Bax increased after treatment with PF-573228 and netrin-1 compared with netrin-1 alone treatment group. Western blotting assays (Fig. 5C, Additional file 6: Fig S5C) were performed to detect the phosphorylation of MAPK transducers and the results showed that PF-573228 significantly inhibited netrin-1-induced phosphorylation of FAK, c-Raf, MEK and ERK. In addition, the ERK inhibitor, Magnolin was applied to treat B-ALL cells. The results of CCK-8 assays (Fig. 5B, Additional file 6: Fig S5B) showed that Magnolin decreased the proliferation of B-ALL cells treated with netrin-1. The apoptosis rates of B-ALL cells cotreated with Magnolin and netrin-1 were significantly increased compared with those of cells treated with netrin-1 alone (Fig. 5H, I, Additional file 6: Fig S5H and 5I). Western blotting assays (Fig. 5E, Additional file 6: Fig S5E) showed that the protein levels of CDK4, PCNA and Bcl-2 decreased while Bax increased after treatment with Magnolin and netrin-1 compared with netrin-1 alone treatment group. In summary, our results demonstrated that ERK-MAPK signaling pathway downstream of FAK was involved in anti-apoptotic effect induced by netrin-1 in B-ALL cells.

## Discussion

In the present study, we firstly observed that netrin-1 expression was increased in B-ALL and that netrin-1 expression was upregulated in patients with high risk and intermediate risk stratification. We then found that netrin-1 induced an anti-apoptotic effect of B-ALL cells, implying netrin-1 plays an oncogenic role in B-ALL. Using co-immunoprecipitation, we showed that netrin-1 induces B-ALL cells anti-apoptotic effects by interacting with the receptor Unc5b and activating the FAK-MAPK signaling pathway. It was also reported that netrin-1 was upregulated in multiple malignancies including glioma [32], breast cancer [26], ovarian malignancies [33], non-small cell lung cancer [34], hepatocellular carcinoma [35], melanoma [36] and medulloblastoma [27] which was consistent with our results. Nevertheless, there were also opposite opinions that netrin-1 expression was decreased in stage I/II pancreatic ductal adenocarcinoma (PDAC) [20], prostate tumors [37] and nearly half of brain tumors [38]. An [20] showed that netrin-1 inhibited 3D growth and decreased the adhesion of PDAC cells but showed no anti-apoptotic effect on PDAC cells. Alain Latil [37] found reduced expression of netrin-1 and DCC in neoplastic prostate tissues showing the disruption of cell–cell contacts. However, we found that netrin-1 induced the anti-apoptotic effect of B-ALL cells, and this effect of netrin-1 was also discovered in lung cancer, advanced neuroblastoma and breast cancer, in which the expression of netrin-1 was also upregulated [39]. Thus, the role of netrin-1 in those tumor tissues with reduced netrin-1 expression might exert a different functional pattern from those with increased expression of netrin-1. And the functional differences of netrin-1 in malignant tumors suggested that the functions and underlying mechanisms of netrin-1 were diverse and complex.

Netrin-1 exerted its biological functions through a variety of receptors [20, 32, 40]. Unc5b is one of the common receptors of netrin-1 and netrin-1 -Unc5b pathway has been involved in PDAC tumors [20], bladder cancer [41] and renal cell carcinoma [42]. In the present study, we found that netrin-1 induced an anti-apoptotic effect by interacting with the receptor Unc5b in B-ALL cells. In the tumorigenesis of colorectal cancer, netrin-1 and its receptors exert carcinogenic effects through two complementary events [43]: one is through overexpression of netrin-1, which induces proliferation on tumor cells, and the other is via the loss of netrin-1 receptors, which results in tumor cell survival. Although the results of our study also showed that recombinant netrin-1 notably reduced B-ALL cell apoptosis by binding with Unc5b, the apoptosis rate of UNC5B interfering cells, however, did not decrease compared with that of vector control cells, which suggested that Unc5b did not function as a typical dependent receptor in B-ALL cells. In the studies of thyroid cancer cells and breast cancer cells [44, 45], reduced Unc5B expression was found to supress the proliferation, migration and invasion of thyroid cancer cells and breast cancer cells, suggesting that Unc5b could not induce apoptosis and function as an oncogene in these cells, which was similar to the findings of this study. Thus, like netrin-1, unc5b might exert different functions in different malignant tumors, and the underlying mechanism needs further investigation.

FAK is one of the main downstream effectors through the receptors DCC and Unc5b [46–48]. In the present study, we observed that the phosphorylation of FAK was upregulated in B-ALL cells under the treatment of recombinant netrin-1 and abolished by Unc5b interference, indicating that FAK was the main downstream effector of Netrin-1-Unc5b binding in B-ALL cells. In addition, our results suggested that netrin-1 exerted its functions by activating MAPK signaling pathway, which was reported to play an essential role in cell proliferation [49]. It was also reported that NTN1 promoted gastric cancer cell proliferation via activation of FAK/ERK/MAPK [50]. However, one previous study in PDAC cells [20]showed that NTN1 suppressed 3D growth of PDAC cells by inhibiting MEK/ERK pathway. However, the underlying mechanism of netrin-1 in PDAC cells in that study was related to adhesion regulation, which was not directly related to apoptosis regulation. Another study [51] also indicated that NTN1 induced inhibition of MEK/ERK pathway in epithelial cells during lung branching morphogenesis, but the tissue was normal epithelium and the receptor in this process was neogenin-1 instead. All these studies suggested that NTN1 may have a bidirectional impact on MEK/ERK pathway, and the cause for this phenomenon might need more thorough investigation.

Netrin-1 functioned in a concentration dependent manner in bioprocesses [52, 53]. In the present study, all concentrations of netrin-1 effectively induced anti-apoptotic effect on B-ALL cells. However, there were different optimal concentrations of netrin-1 for REH cells and SUP-B15 cells, which were 100 ng/ml and 50 ng/ml, respectively. Additionally, we noticed that the median concentration of netrin-1 in the B-ALL patients was 98.84 ng/ml, which was also in the interval between 50 and 100 ng/mL. These results suggested that netrin-1 concentration in this interval might be oncogenic for B cells and that the concentration of netrin-1 might have the potentiality to be applied to evaluate the risk of B-ALL tumorigenesis.

Netrin-1 promotes the motility of many types of tumor, such as PDAC cells [54], hepatocellular carcinoma cells [35] and tumors of the nervous system [25]. It is believed that upregulation of Netrin-1 may promote epithelial-mesenchymal transition (EMT), thus contributing to invasion [35]. The netrin-1-FAK axis was considered as an inducer of the migration and adhesion of many tumor cells [55, 56]. In this study, we found FAK activated by netrin-1 promoted the anti-apoptotic effect while no effect on the migration ability of B-ALL cell lines. Additionally, FAK was found to support leukemia cell survival in acute myeloid leukemia [57]. These results suggested a difference in the function of netrin-1-FAK axis between hematopoietic malignancies and the solid tumors, which might root in the microenvironmental difference between these two types.

However, there are still some limitations in our study. First, we did not detect the phosphorylation levels of FAK in B-ALL patients. Second, an in vitro assay demonstrated that netrin-1 induced anti-apoptotic effect on B-ALL cells, but the findings have yet to be confirmed in vivo. As a next step, we will verify the relevance of netrin-1 concentration and phosphorylation level of FAK in the collected clinical specimens, and construct B-ALL murine models to clarify the anti-apoptotic effect of netrin-1 on B-ALL in vivo.

## Conclusions

In conclusion, our study demonstrated that netrin-1 functions as a novel inducer of anti-apoptosis in B-ALL cells, which was mediated by abnormal activation of FAK-MAPK pathway via the receptor Unc5b. The therapeutic potential of targeting Netrin-1/Unc5b-FAK-MAPK signaling pathway in B-ALL might need more evaluation and investigation.

## Supplementary Information

**Additional file 1**: **Table S1**. Primers used in real-time PCR analysis.

**Additional file 2**: **Fig. S2**. Netrin-1 induced the anti-apoptotic effect of SUP-B15 cells. **A** The 450 nm absorbance of SUP-B15 cells treated with exogenous recombinant netrin-1 in concentration ladder of 0,25,50,100 ng/ml in 3 days (*n*=3, *P < 0.05, **P < 0.01, ***P < 0.001). **B** The expression of CDK4, PCNA, Bcl-2 and Bax in SUP-B15 cells treated with exogenous netrin-1 in concentration ladder (0,25,50,100 ng/ml, 24h) detected by western blotting. The expression of Gapdh was applied as an internal control. **C** Flow cytometric analysis of apoptotic ratio of SUP-B15 cells treated with exogenous recombinant netrin-1 in concentration ladder of 0, 25, 50 and 100 ng/ml after 24 hours. The early apoptotic cells were marked with positive Annexin V staining (APC-A) and negative 7-AAD staining. **D** Column graph of the early and late apoptosis ratio of SUP-B15 cells treated with exogenous recombinant netrin-1 in concentration ladder of 0,25,50 and 100 ng/ml after 24 hours detected by flow cytometric analysis (*n *= 3, *P < 0.05).

**Additional file 3**: **Fig. S1**. Netrin-1 has no effect on B-ALL cells migrating. **A** The transwell analysis of migration of REH cells treated with netrin-1 in concentration ladder of 0,25,50 and 100 ng/ml after 24 hours. The migrated cells were stained with crystal violet (*n* = 3). **B** Column graphs of migratory cell counts of REH cells treated with netrin-1 in concentration ladder of 0,25,50 and 100 ng/ml. **C** The transwell analysis of migration of SUP-B15 cells treated with netrin-1 in concentration ladder of 0,25,50 and 100 ng/ml after 24 hours(*n *= 3). The migrated cells were stained with crystal violet. **D** Column graphs of migratory cell counts of SUP-B15 cells treated with netrin-1 in concentration ladder of 0,25,50 and 100 ng/ml.

**Additional file 4**: **Fig. S3**. Netrin-1 induced the anti-apoptotic effect of REH cells through the Unc5b receptor. **A** Real-time PCR analysis of the expression of netrin-1 receptor in SUP-B15 cells. ACTIN was used as an internal control (*n* = 3). **B** An anti-His-tag antibody was used to pull down the histagged netrin-1 protein after exogenous recombinant netrin-1 treatment, followed by immunoblotting analysis of the receptor and netrin-1 levels in the precipitation. **C** The expression of Unc5b was efficiently decreased following transfection with UNC5B interference lentivirus in SUP-B15 cells. **D** The 450 nm absorbance of the 3 day growth curve of shCtrl cells, shCtrl cells treated with netrin-1(100 ng/ml), shUNC5B cells and shUNC5B cells treated with netrin-1(*n *= 3, **P < 0.01, ***P < 0.001, ****P < 0.0001). **E** The expression levels of CDK4, PCNA, Bcl-2 and Bax in shCtrl cells, shCtrl cells treated with netrin-1 alone (100 ng/ml, 24h), shUNC5B cells and shUNC5B cells treated with netrin-1. The expression level was detected by western blotting. The expression of Gapdh was applied as an internal control. **F** Flow cytometric analysis of the apoptotic ratio of shCtrl cells, shCtrl cells treated with netrin-1 alone (100 ng/ml, 24h), shUNC5B cells and shUNC5B cells treated with netrin-1. **G** Column graph of the early apoptotic ratio of shCtrl cells , shCtrl cells treated with netrin-1 alone, shUNC5B cells and shUNC5B cells treated with netrin-1 (*n *= 3,*P < 0.05, **P < 0.01).

**Additional file 5**: **Fig. S4**. Netrin-1 increased the phosphorylation of FAK-MAPK pathway in REH cells. **A** The total expression level and phosphorylation level of FAK, c-Raf, Mek1/2 and Erk1/2 in SUP-B15 cells treated with exogenous netrin-1 in concentration ladder (0,25,50,100 ng/ml, 30 min). The expression level was detected by western blotting. The expression level of Gapdh was applied as an internal control. **B** Interference of UNC5B expression decreased the phosphorylation of FAK-MAPK pathway in SUP-B15 cells. The total protein expression level and phosphorylation level of FAK, c-Raf, Mek1/2 and Erk1/2 in shCtrl cells, shCtrl cells treated with netrin-1 alone (100 ng/ml, 30 min), shUNC5B cells and shUNC5B cells treated with netrin-1 were detected by western blotting assay. The expression level of Gapdh was applied as an internal control.

**Additional file 6**: **Fig. S5**. inhibition of FAK and Erk 1/2 could reduce SUP-B15 cells survival. **A** The 450 nm absorbance of the 3 day growth curve of SUP-B15 cells (ctrl) , SUP-B15 cells treated with netrin-1 alone(100 ng/ml), SUP-B15 cells treated with PF-573228 alone (20 nM)and SUP-B15 cells co-treated with netrin-1 and PF-573228 (*n *= 3, **P < 0.01, ***P < 0.001, ****P < 0.0001). **B** The 450 nm absorbance of the 3 day growth curve of SUP-B15 cells (ctrl), SUP-B15 cells treated with netrin-1 alone(100 ng/ml), SUP-B15 cells treated with Magnolin alone (87 nM)and SUP-B15 cells co-treated with netrin-1 and Magnolin (*n *= 3, **P < 0.01, ****P < 0.0001). **C** The total protein expression level and phosphorylation level of FAK, c-Raf, Mek1/2 and Erk1/2 in SUP-B15 cells treated with netrin-1 alone (100 ng/ml, 24h) and SUP-B15 cells co-treated with netrin-1 and PF-573228(20 nM, 24h). The expression level of Gapdh was applied as an internal control. **D** The expression levels of CDK4, PCNA, Bcl-2 and Bax in SUP-B15 cells (ctrl), SUP-B15 cells treated with netrin-1 alone (100 ng/ml, 24h) (20 nM, 24h), SUP-B15 cells treated with PF-573228 alone (20 nM, 24h) and SUP-B15 cells co-treated with netrin-1 and PF-573228 were detected by western blotting. The expression level of Gapdh was applied as an internal control. **E** The expression levels of CDK4, PCNA, Bcl-2 and Bax in SUP-B15 cells (ctrl), SUP-B15 cells treated with netrin-1 alone (100 ng/ml, 24h), SUP-B15 cells treated with Magnolin alone (87 nM, 24h) and SUP-B15 cells co-treated with netrin-1 and Magnolin were detected by western blotting. The expression level of Gapdh was applied as internal control. **F** Flow cytometric analysis of apoptotic ratio of SUP-B15 cells (ctrl), SUP-B15 cells treated with netrin-1 alone (100 ng/ml, 24h), SUP-B15 cells treated with PF-573228 alone (20 nM, 24h) and SUP-B15 cells co-treated with netrin-1 and PF-573228. **G** Column graph of the early apoptotic ratio of SUP-B15 cells (ctrl), SUP-B15 cells treated with netrin-1 alone, SUP-B15 cells treated with PF-573228 alone and SUP-B15 cells co-treated with netrin-1 and PF-573228(*n *= 3, *P < 0.05, **P < 0.01). **H** Flow cytometric analysis of apoptotic ratio of SUP-B15 cells (ctrl), SUP-B15 cells treated with netrin-1 alone (100 ng/ml, 24h), SUP-B15 cells treated with Magnolin (87 nM, 24h) and SUP-B15 cells co-treated with netrin-1 and Magnolin. **I** Column graph of the early apoptotic ratio of SUP-B15 cells (ctrl), SUP-B15 cells treated with netrin-1 alone, SUP-B15 cells treated with Magnolin alone and SUP-B15 cells co-treated with netrin-1 and Magnolin (*n *= 3, *P < 0.05).

## Data Availability

The data that support the findings of this study are available from the corresponding author upon reasonable request.

## Abbreviations

B-ALL: B-cell acute lymphoblastic leukemia
ELISA: Enzyme-linked immunosorbent assay
co-IP: Co-immunoprecipitation
FAK: Focal adhesion kinase
MAPK: Mitogen activated protein kinase
PDAC: Pancreatic ductal adenocarcinoma
