# Supplementary material for: Netrin-1 induces the anti-apoptotic and pro-survival effects of B-ALL cells through the Unc5b-MAPK axis
Source: Cell Commun Signal. 2022 Aug 16;20:122. doi: 10.1186/s12964-022-00935-y (PMC9380321; doi:10.1186/s12964-022-00935-y)
Supplement: Supplementary file 2 — Additional file 1: Table S1. Primers used in real-time PCR analysis. [file 12964_2022_935_MOESM2_ESM.docx]

Supplementary Material

Table1 primers used in real-time PCR analysis

| Gene symbol | Primer Sequences (5’-3’) | Template Sequences |
| --- | --- | --- |
| DCC | Forward: ACCCAAGCTGGCTTTTGTACT  Reverse: TGTGACGGCATCAGAAGGTTC | NM_005215.3 |
| NEO-1 | Forward: ACCTTCCTCAGTTTATGCTGGG  Reverse: ACTTTCCACTACGCAGCGATA | NM_001172623.1 |
| UNC5A | Forward: GGACACCCGCAACTGTACC  Reverse: AATGGACGAGTCAGCCACATC | NM_133369.2 |
| UNC5B | Forward: GCAGGACTCCACCGACAAAAT  Reverse: GCGAAATCGCTAGGGTATGTG | NM_170744.4 |
| UNC5C | Forward: TGGGACTGGGATACTTGCTG  Reverse: ACAGTACAGGTTCACAGGCTTAT | NM_003728.3 |
| UNC5D | Forward: TGGGTCCATCAGAACGAGC  Reverse: CCACCTGTTGCCTAGTAACATTG | NM_080872.2 |
| ITGA3 | Forward: TGTGGCTTGGAGTGACTGTG  Reverse: TCATTGCCTCGCACGTAGC | NM_005501.2 |
| ITGB1 | Forward: ATGACAGAAGGGAGTTTGC  Reverse: TTGACCACAGTTGTTACGG | NM_002211.3 |
| ITGB4 | Forward: ACTGGGTCCTTTCACATCCG  Reverse: TGTTGCCCTTCTGGTCCTCC | NM_000213.3 |

**Supplementary Figure 1 Netrin-1 has no effect on B-ALL cells migrating.**

1. The transwell analysis of migration of REH cells treated with netrin-1 in concentration ladder of 0,25,50 and 100 ng/ml after 24 hours.The migrated cells were stained with crystal violet (n=3). **(B)** Column graphs of migratory cell counts of REH cells treated with netrin-1 in concentration ladder of 0,25,50 and 100 ng/ml. **(C)** The transwell analysis of migration of SUP-B15 cells treated with netrin-1 in concentration ladder of 0,25,50 and 100 ng/ml after 24 hours(n=3). The migrated cells were stained with crystal violet. **(D)** Column graphs of migratory cell counts of SUP-B15 cells treated with netrin-1 in concentration ladder of 0,25,50 and 100 ng/ml.

**Supplementary Figure 2 Netrin-1 induced the anti-apoptotic effect of SUP-B15 cells.**

1. The 450 nm absorbance of SUP-B15 cells treated with exogenous recombinant netrin-1 in concentration ladder of 0.25,50,100 ng/ml in 3 days (n=3, *P<0.05, **P<0.01, ***P<0.001**). (B)** The expression of CDK4, PCNA, Bcl-2 and Bax in SUP-B15 cells treated with exogenous netrin-1 in concentration ladder (0,25,50,100 ng/ml, 24h) detected by western blotting. The expression of Gapdh was applied as an internal control. **(C)** Flow cytometric analysis of apoptotic ratio of SUP-B15 cells treated with exogenous recombinant netrin-1 in concentration ladder of 0, 25, 50 and 100 ng/ml after 24 hours. The early apoptotic cells were marked with positive Annexin V staining (APC-A) and negative 7-AAD staining. **(D)** Column graph of the early and late apoptosis ratio of SUP-B15 cells treated with exogenous recombinant netrin-1 in concentration ladder of 0,25,50 and 100 ng/ml after 24 hours detected by flow cytometric analysis (n=3, *P<0.05).

**Supplementary Figure 3 Netrin-1 induced the anti-apoptotic effect of REH cells through the Unc5b receptor**

(A) Real-time PCR analysis of the expression of netrin-1 receptor in SUP-B15 cells. ACTIN was used as an internal control (n=3). **(B)** An anti-His-tag antibody was used to pull down the histagged netrin-1 protein after exogenous recombinant netrin-1 treatment, followed by immunoblotting analysis of the receptor and netrin-1 levels in the precipitation. **(C)** The expression of Unc5b was efficiently decreased following transfection with UNC5B interference lentivirus in SUP-B15 cells.  **(D)** The 450 nm absorbance of the 3 day growth curve of shCtrl cells, shCtrl cells treated with netrin-1(100 ng/ml), shUNC5B cells and shUNC5B cells treated with netrin-1(n=3, **P<0.01, ***P<0.001, ****P<0.0001). **(E)** The expression levels of CDK4, PCNA, Bcl-2 and Bax in shCtrl cells, shCtrl cells treated with netrin-1 alone (100 ng/ml, 24h), shUNC5B cells and shUNC5B cells treated with netrin-1. The expression level was detected by western blotting. The expression of Gapdh was applied as an internal control. **(F)**Flow cytometric analysis of the apoptotic ratio of shCtrl cells, shCtrl cells treated with netrin-1 alone (100 ng/ml, 24h), shUNC5B cells and shUNC5B cells treated with netrin-1. **（G）**Column graph of the early apoptotic ratio of shCtrl cells , shCtrl cells treated with netrin-1 alone, shUNC5B cells and shUNC5B cells treated with netrin-1 (n=3,*P<0.05, **P<0.01).

**Supplementary Figure 4 Netrin-1 increased the phosphorylation of FAK-MAPK pathway in REH cells.**

1. The total expression level and phosphorylation level of FAK, c-Raf, Mek1/2 and Erk1/2 in SUP-B15 cells treated with exogenous netrin-1 in concentration ladder (0,25,50,100 ng/ml, 30 min). The expression level was detected by western blotting. The expression level of Gapdh was applied as an internal control. **(B)** Interference of UNC5B expression decreased the phosphorylation of FAK-MAPK pathway in SUP-B15 cells. The total protein expression level and phosphorylation level of FAK, c-Raf, Mek1/2 and Erk1/2 in shCtrl cells, shCtrl cells treated with netrin-1 alone (100 ng/ml, 30 min), shUNC5B cells and shUNC5B cells treated with netrin-1 were detected by western blotting assay. The expression level of Gapdh was applied as an internal control

**Supplementary Figure5 nhibition of FAK and Erk 1/2 could reduce SUP-B15 cells survival.**

**(A)** The 450 nm absorbance of the 3 day growth curve of SUP-B15 cells (ctrl) , SUP-B15 cells treated with netrin-1 alone(100 ng/ml), SUP-B15 cells treated with PF-573228 alone (20 nM)and SUP-B15 cells co-treated with netrin-1 and PF-573228 (n=3, **P<0.01, ***P<0.001, ****P<0.0001).**(B)** The 450 nm absorbance of the 3 day growth curve of SUP-B15 cells (ctrl) , SUP-B15 cells treated with netrin-1 alone(100 ng/ml), SUP-B15 cells treated with Magnolin alone (87 nM)and SUP-B15 cells co-treated with netrin-1 and Magnolin (n=3, **P<0.01, ****P<0.0001). **(C)** The total protein expression level and phosphorylation level of FAK, c-Raf, Mek1/2 and Erk1/2 in SUP-B15 cells treated with netrin-1 alone (100 ng/ml, 24h) and SUP-B15 cells co-treated with netrin-1 and PF-573228(20 nM, 24h). The expression level of Gapdh was applied as an internal control. **(D)** The expression levels of CDK4, PCNA, Bcl-2 and Bax in SUP-B15 cells (ctrl), SUP-B15 cells treated with netrin-1 alone (100 ng/ml, 24h) (20 nM, 24h), SUP-B15 cells treated with PF-573228 alone (20 nM, 24h) and SUP-B15 cells co-treated with netrin-1 and PF-573228 were detected by western blotting. The expression level of Gapdh was applied as an internal control. **(E)** The expression levels of CDK4, PCNA, Bcl-2 and Bax in SUP-B15 cells (ctrl), SUP-B15 cells treated with netrin-1 alone (100 ng/ml, 24h), SUP-B15 cells treated with Magnolin alone (87 nM, 24h) and SUP-B15 cells co-treated with netrin-1 and Magnolin were detected by western blotting. The expression level of Gapdh was applied as internal control.

**(F)**Flow cytometric analysis of apoptotic ratio of SUP-B15 cells (ctrl), SUP-B15 cells treated with netrin-1 alone (100 ng/ml, 24h), SUP-B15 cells treated with PF-573228 alone (20 nM, 24h) and SUP-B15 cells co-treated with netrin-1 and PF-573228. **(G)** Column graph of the early apoptotic ratio of SUP-B15 cells (ctrl), SUP-B15 cells treated with netrin-1 alone, SUP-B15 cells treated with PF-573228 alone and SUP-B15 cells co-treated with netrin-1 and PF-573228(n=3, *P<0.05, **P<0.01). **(H)** Flow cytometric analysis of apoptotic ratio of SUP-B15 cells (ctrl), SUP-B15 cells treated with netrin-1 alone (100 ng/ml, 24h), SUP-B15 cells treated with Magnolin (87 nM, 24h) and SUP-B15 cells co-treated with netrin-1 and Magnolin. **(I)** Column graph of the early apoptotic ratio of SUP-B15 cells (ctrl), SUP-B15 cells treated with netrin-1 alone, SUP-B15 cells treated with Magnolin alone and SUP-B15 cells co-treated with netrin-1 and Magnolin (n=3, *P<0.05).
