# Supplementary figures and images for: Netrin-1 induces the anti-apoptotic and pro-survival effects of B-ALL cells through the Unc5b-MAPK axis
Source: Cell Commun Signal. 2022 Aug 16;20:122. doi: 10.1186/s12964-022-00935-y (PMC9380321; doi:10.1186/s12964-022-00935-y)

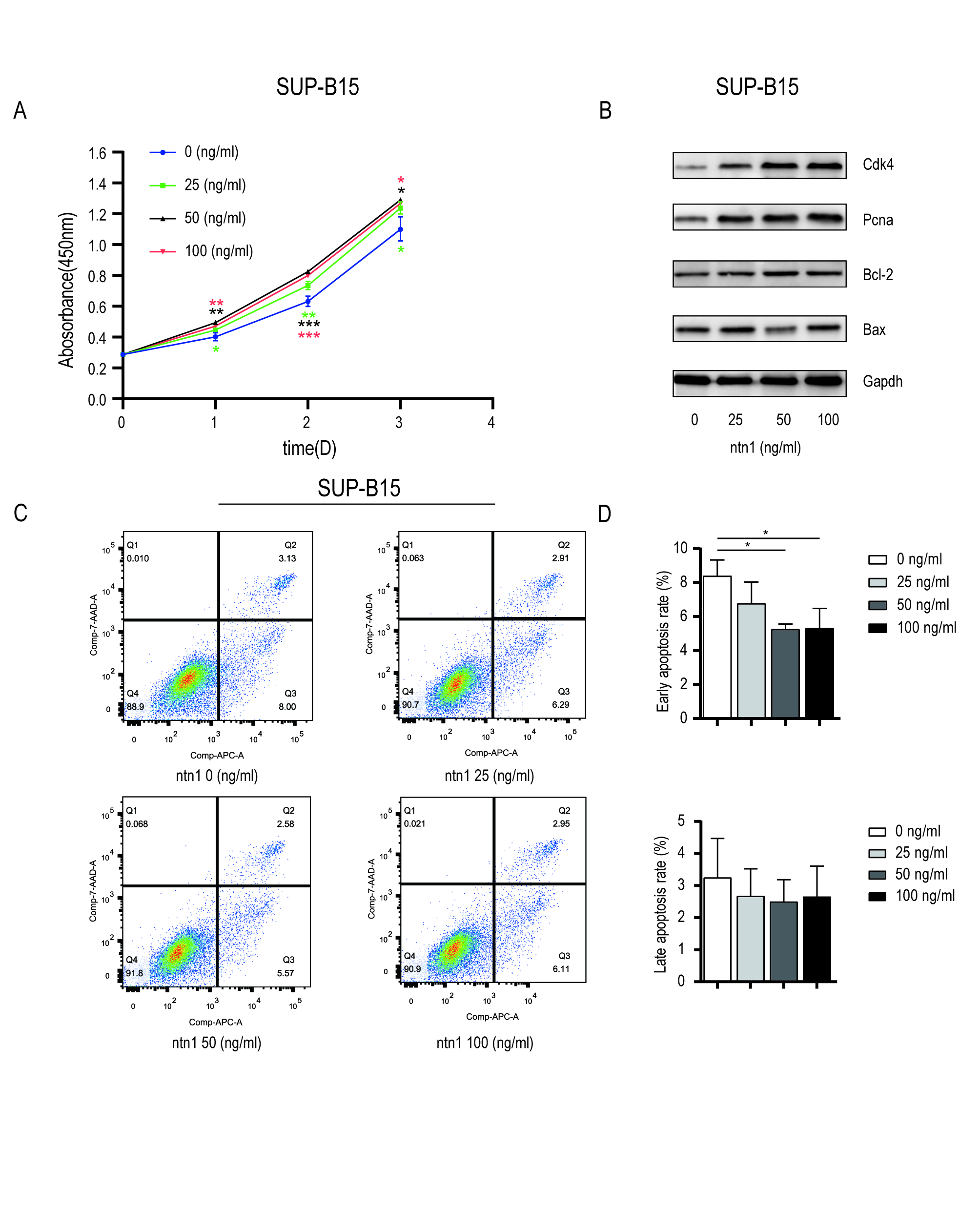

Supplement: Supplementary file 3 — Additional file 2: Fig. S2. Netrin-1 induced the anti-apoptotic effect of SUP-B15 cells. A The 450 nm absorbance of SUP-B15 cells treated with exogenous recombinant netrin-1 in concentration ladder of 0,25,50,100 ng/ml in 3 days (n=3, *P < 0.05, **P < 0.01, ***P < 0.001). B The expression of CDK4, PCNA, Bcl-2 and Bax in SUP-B15 cells treated with exogenous netrin-1 in concentration ladder (0,25,50,100 ng/ml, 24h) detected by western blotting. The expression of Gapdh was applied as an internal control. C Flow cytometric analysis of apoptotic ratio of SUP-B15 cells treated with exogenous recombinant netrin-1 in concentration ladder of 0, 25, 50 and 100 ng/ml after 24 hours. The early apoptotic cells were marked with positive Annexin V staining (APC-A) and negative 7-AAD staining. D Column graph of the early and late apoptosis ratio of SUP-B15 cells treated with exogenous recombinant netrin-1 in concentration ladder of 0,25,50 and 100 ng/ml after 24 hours detected by flow cytometric analysis (n = 3, *P < 0.05). [file 12964_2022_935_MOESM3_ESM.tif]

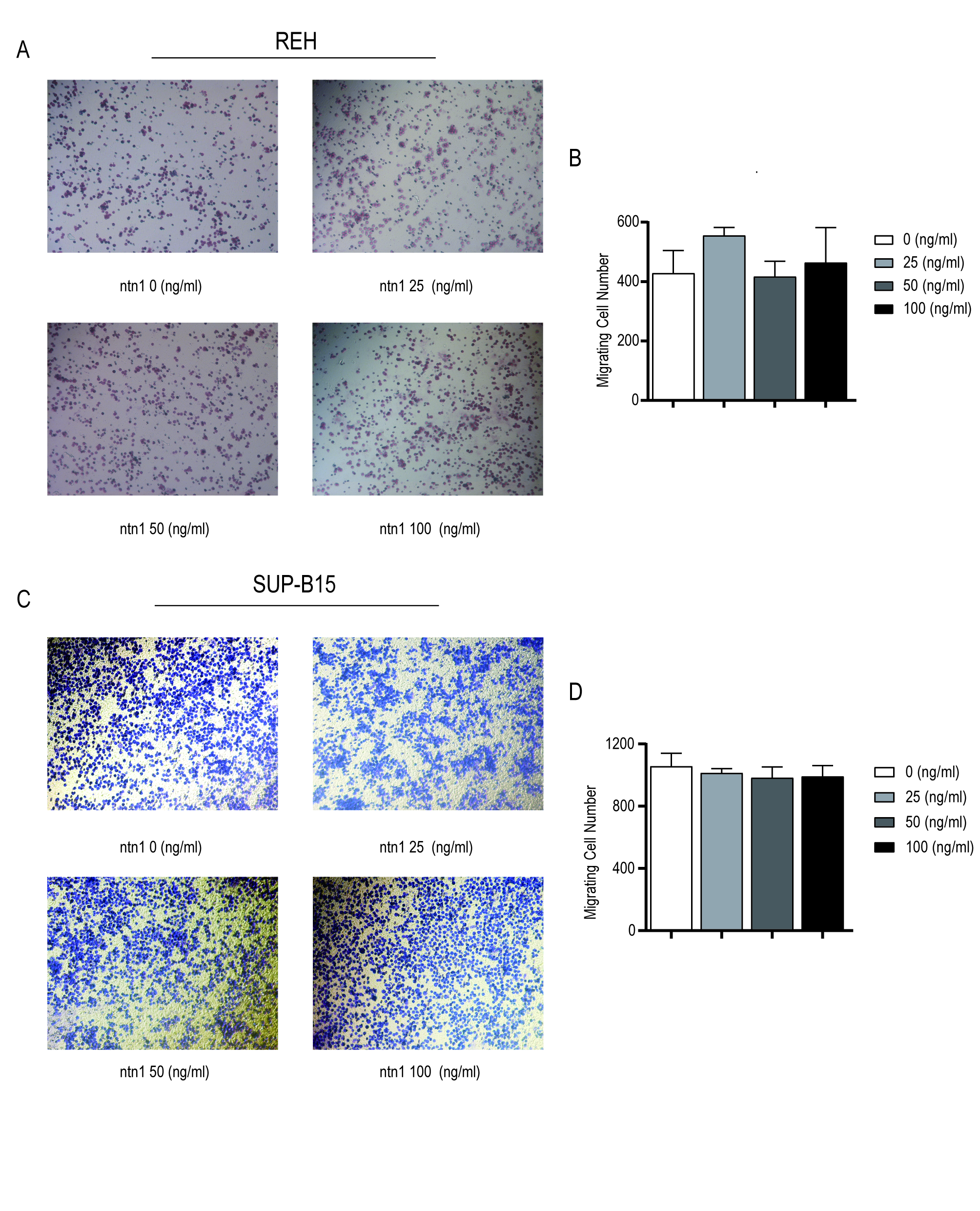

Supplement: Supplementary file 4 — Additional file 3: Fig. S1. Netrin-1 has no effect on B-ALL cells migrating. A The transwell analysis of migration of REH cells treated with netrin-1 in concentration ladder of 0,25,50 and 100 ng/ml after 24 hours. The migrated cells were stained with crystal violet (n = 3). B Column graphs of migratory cell counts of REH cells treated with netrin-1 in concentration ladder of 0,25,50 and 100 ng/ml. C The transwell analysis of migration of SUP-B15 cells treated with netrin-1 in concentration ladder of 0,25,50 and 100 ng/ml after 24 hours(n = 3). The migrated cells were stained with crystal violet. D Column graphs of migratory cell counts of SUP-B15 cells treated with netrin-1 in concentration ladder of 0,25,50 and 100 ng/ml. [file 12964_2022_935_MOESM4_ESM.tif]

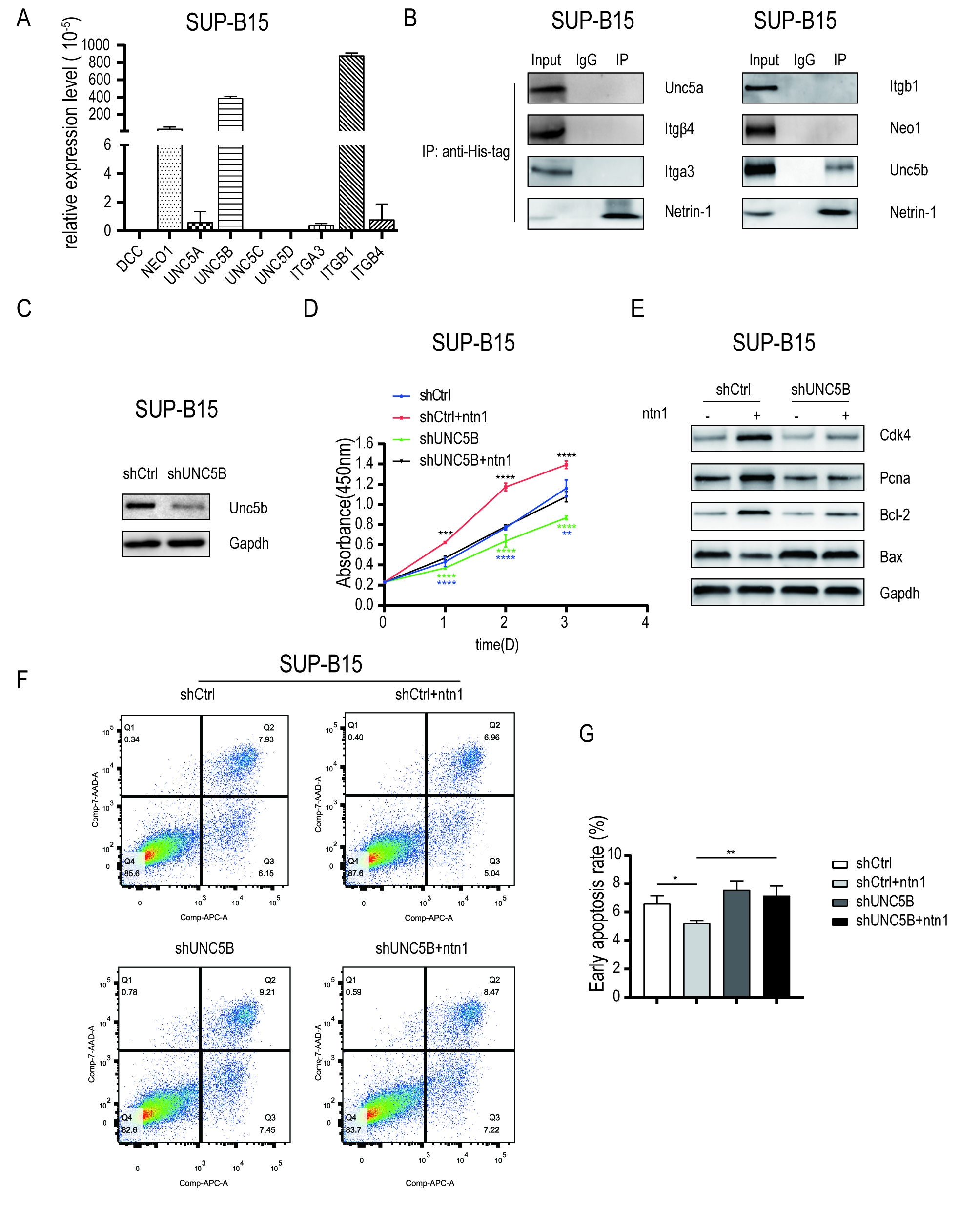

Supplement: Supplementary file 5 — Additional file 4: Fig. S3. Netrin-1 induced the anti-apoptotic effect of REH cells through the Unc5b receptor. A Real-time PCR analysis of the expression of netrin-1 receptor in SUP-B15 cells. ACTIN was used as an internal control (n = 3). B An anti-His-tag antibody was used to pull down the histagged netrin-1 protein after exogenous recombinant netrin-1 treatment, followed by immunoblotting analysis of the receptor and netrin-1 levels in the precipitation. C The expression of Unc5b was efficiently decreased following transfection with UNC5B interference lentivirus in SUP-B15 cells. D The 450 nm absorbance of the 3 day growth curve of shCtrl cells, shCtrl cells treated with netrin-1(100 ng/ml), shUNC5B cells and shUNC5B cells treated with netrin-1(n = 3, **P < 0.01, ***P < 0.001, ****P < 0.0001). E The expression levels of CDK4, PCNA, Bcl-2 and Bax in shCtrl cells, shCtrl cells treated with netrin-1 alone (100 ng/ml, 24h), shUNC5B cells and shUNC5B cells treated with netrin-1. The expression level was detected by western blotting. The expression of Gapdh was applied as an internal control. F Flow cytometric analysis of the apoptotic ratio of shCtrl cells, shCtrl cells treated with netrin-1 alone (100 ng/ml, 24h), shUNC5B cells and shUNC5B cells treated with netrin-1. G Column graph of the early apoptotic ratio of shCtrl cells , shCtrl cells treated with netrin-1 alone, shUNC5B cells and shUNC5B cells treated with netrin-1 (n = 3,*P < 0.05, **P < 0.01). [file 12964_2022_935_MOESM5_ESM.tif]

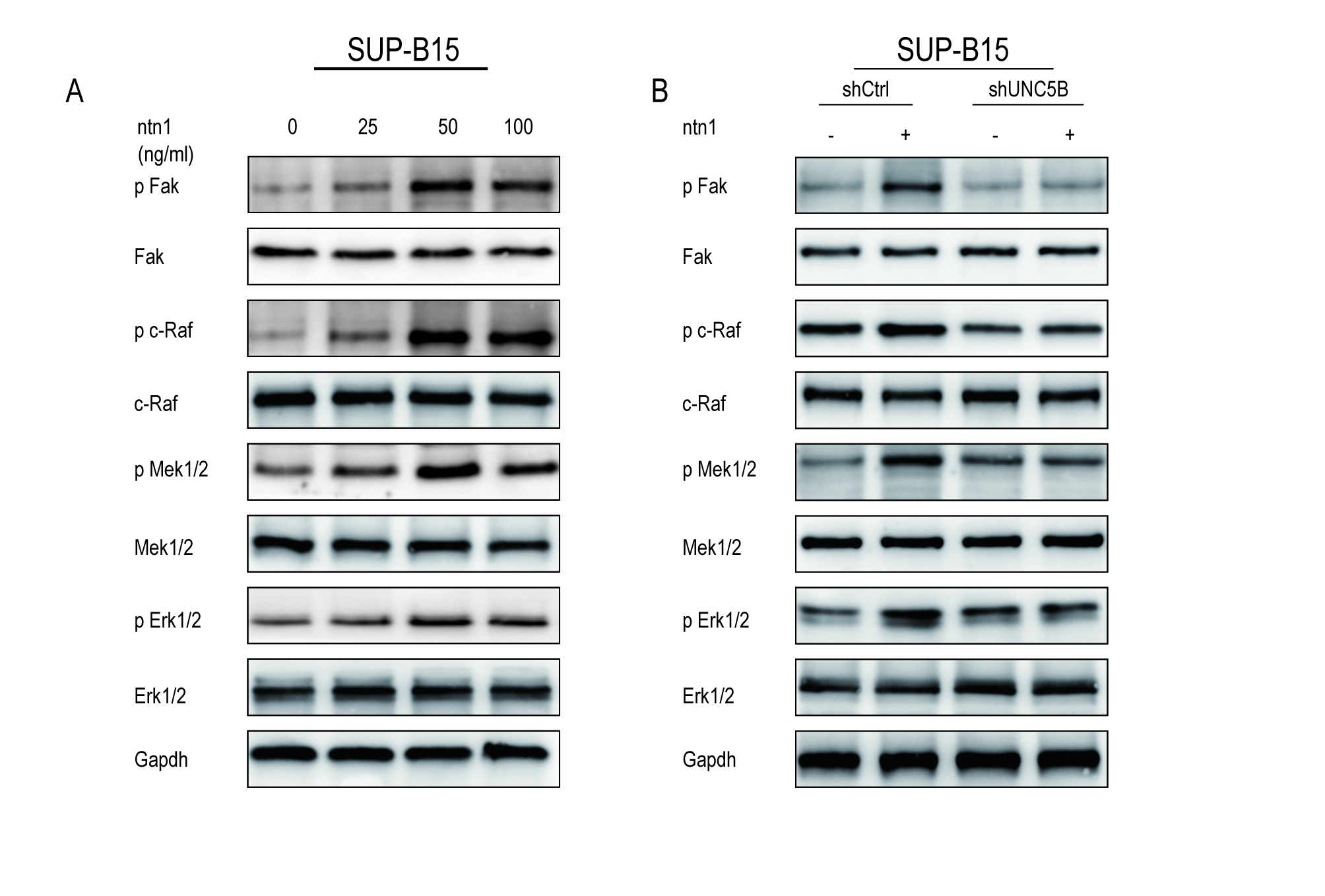

Supplement: Supplementary file 6 — Additional file 5: Fig. S4. Netrin-1 increased the phosphorylation of FAK-MAPK pathway in REH cells. A The total expression level and phosphorylation level of FAK, c-Raf, Mek1/2 and Erk1/2 in SUP-B15 cells treated with exogenous netrin-1 in concentration ladder (0,25,50,100 ng/ml, 30 min). The expression level was detected by western blotting. The expression level of Gapdh was applied as an internal control. B Interference of UNC5B expression decreased the phosphorylation of FAK-MAPK pathway in SUP-B15 cells. The total protein expression level and phosphorylation level of FAK, c-Raf, Mek1/2 and Erk1/2 in shCtrl cells, shCtrl cells treated with netrin-1 alone (100 ng/ml, 30 min), shUNC5B cells and shUNC5B cells treated with netrin-1 were detected by western blotting assay. The expression level of Gapdh was applied as an internal control. [file 12964_2022_935_MOESM6_ESM.tif]

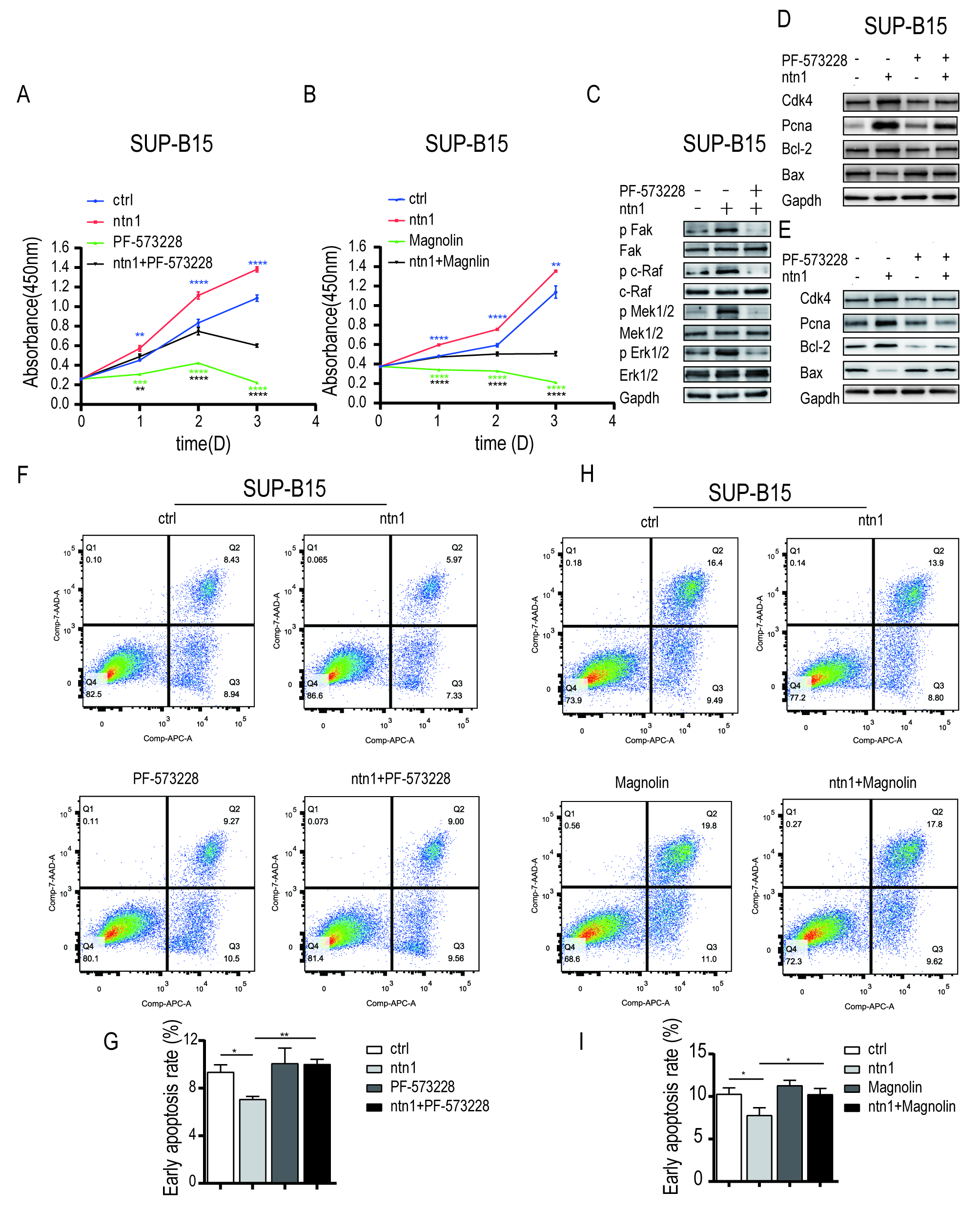

Supplement: Supplementary file 7 — Additional file 6: Fig. S5. inhibition of FAK and Erk 1/2 could reduce SUP-B15 cells survival. A The 450 nm absorbance of the 3 day growth curve of SUP-B15 cells (ctrl) , SUP-B15 cells treated with netrin-1 alone(100 ng/ml), SUP-B15 cells treated with PF-573228 alone (20 nM)and SUP-B15 cells co-treated with netrin-1 and PF-573228 (n = 3, **P < 0.01, ***P < 0.001, ****P < 0.0001). B The 450 nm absorbance of the 3 day growth curve of SUP-B15 cells (ctrl), SUP-B15 cells treated with netrin-1 alone(100 ng/ml), SUP-B15 cells treated with Magnolin alone (87 nM)and SUP-B15 cells co-treated with netrin-1 and Magnolin (n = 3, **P < 0.01, ****P < 0.0001). C The total protein expression level and phosphorylation level of FAK, c-Raf, Mek1/2 and Erk1/2 in SUP-B15 cells treated with netrin-1 alone (100 ng/ml, 24h) and SUP-B15 cells co-treated with netrin-1 and PF-573228(20 nM, 24h). The expression level of Gapdh was applied as an internal control. D The expression levels of CDK4, PCNA, Bcl-2 and Bax in SUP-B15 cells (ctrl), SUP-B15 cells treated with netrin-1 alone (100 ng/ml, 24h) (20 nM, 24h), SUP-B15 cells treated with PF-573228 alone (20 nM, 24h) and SUP-B15 cells co-treated with netrin-1 and PF-573228 were detected by western blotting. The expression level of Gapdh was applied as an internal control. E The expression levels of CDK4, PCNA, Bcl-2 and Bax in SUP-B15 cells (ctrl), SUP-B15 cells treated with netrin-1 alone (100 ng/ml, 24h), SUP-B15 cells treated with Magnolin alone (87 nM, 24h) and SUP-B15 cells co-treated with netrin-1 and Magnolin were detected by western blotting. The expression level of Gapdh was applied as internal control. F Flow cytometric analysis of apoptotic ratio of SUP-B15 cells (ctrl), SUP-B15 cells treated with netrin-1 alone (100 ng/ml, 24h), SUP-B15 cells treated with PF-573228 alone (20 nM, 24h) and SUP-B15 cells co-treated with netrin-1 and PF-573228. G Column graph of the early apoptotic ratio of SUP-B15 cells (ctrl), SUP-B15 cells treated with [file 12964_2022_935_MOESM7_ESM.tif]
